# High-throughput kinase inhibitor screening reveals roles for Aurora and Nuak kinases in neurite initiation and dendritic branching

**DOI:** 10.1038/s41598-021-87521-3

**Published:** 2021-04-14

**Authors:** Sara M. Blazejewski, Sarah A. Bennison, Xiaonan Liu, Kazuhito Toyo-oka

**Affiliations:** Department of Neurobiology and Anatomy, Drexel University College of Medicine, Philadelphia, PA 19129 USA

**Keywords:** High-throughput screening, Imaging, Microscopy, Neuroscience, Cellular neuroscience, Development of the nervous system, Molecular neuroscience, Morphogenesis

## Abstract

Kinases are essential regulators of a variety of cellular signaling processes, including neurite formation—a foundational step in neurodevelopment. Aberrant axonal sprouting and failed regeneration of injured axons are associated with conditions like traumatic injury, neurodegenerative disease, and seizures. Investigating the mechanisms underlying neurite formation will allow for identification of potential therapeutics. We used a kinase inhibitor library to screen 493 kinase inhibitors and observed that 45% impacted neuritogenesis in Neuro2a (N-2a) cells. Based on the screening, we further investigated the roles of Aurora kinases A, B, and C and Nuak kinases 1 and 2. The roles of Aurora and Nuak kinases have not been thoroughly studied in the nervous system. Inhibition or overexpression of Aurora and Nuak kinases in primary cortical neurons resulted in various neuromorphological defects, with Aurora A regulating neurite initiation, Aurora B and C regulating neurite initiation and elongation, all Aurora kinases regulating arborization, and all Nuak kinases regulating neurite initiation and elongation and arborization. Our high-throughput screening and analysis of Aurora and Nuak kinases revealed their functions and may contribute to the identification of therapeutics.

## Introduction

Neurite formation is a highly regulated foundational step in neuromorphogenesis that is closely followed by specification of axons and dendrites and establishment of synaptic connections. It is essential that neurite formation occurs with fidelity to allow the subsequent stages of neuromorphogenesis to be properly completed, such as dendritic branching and synapse formation. Overall neuronal morphology can impact connectivity, which affects the amount of inputs received by a neuron and a neuron’s ability to fire action potentials^1,2^. Neurite formation may be divided into neurite initiation and neurite elongation. Neurite initiation can be associated with a break in the morphological symmetry of a neuron, which drives neuronal polarization. The hallmark of neurite initiation is the extension of actin-rich filopodia and lamellipodia that develop into neurites. These relatively dynamic actin-rich precursor structures are then stabilized via microtubule invasion, which is followed by rapid condensation of the structure to prevent collapse^3^. Once the neurite has been stabilized, neurite elongation will begin as neurites extend and develop growth cones, which are specialized, dynamic regions of actin-rich lamellipodia and filopodia found at the distal ends of all neurites that are critical for forming synaptic contacts and developing neural circuits^4,5^. Neurite elongation is an extremely dynamic process, which is critical for synaptic plasticity, synaptic pruning, and synapse formation, as well as correct pathfinding^6^.

Understanding how neurite formation occurs and is regulated during normal development is necessary for developing therapeutic innovation to either restore neurite formation or trim back aberrant neurite formation. Such treatments would benefit patients suffering from seizures, spinal cord injury, and neurodegenerative disorders that result in axonal injury or degeneration. Neurite formation is controlled by innumerable signaling pathways, with kinases playing an integral role by regulating the phosphorylation status of key target molecules that drive neurite initiation and neurite elongation^7^. Phosphorylation allows for the activation and deactivation of components of a regulatory signaling pathway. Kinases play crucial roles in wide-spread cellular processes including cell cycle regulation, proliferation, metabolism, and apoptosis^8,9^. There is huge therapeutic potential for kinase inhibitors, as nearly 50 kinase inhibitors have already been approved by the FDA for use^10^. The pharmaceutical industry has recently shifted towards expanding the applicability of pre-approved drugs to reduce drug development time by finding additional uses for drugs that already have established safety profiles. Therefore, a better understanding of the role of kinases in regulating neurite formation may contribute to a speedy transition from basic research to clinical treatment due to the prevalence of FDA approved kinase inhibitors.

The kinase signaling pathways that coordinate neurite formation regulate a variety of processes such as cytoskeletal rearrangement and interaction between actin and microtubules, addition to the plasma membrane, cell adhesion, protein synthesis, and coordination between the cytoskeleton and plasma membrane^11^. These processes need to be precisely regulated to cause the morphological changes that occur during neurite formation. Regulation via protein kinases is advantageous because changes in phosphorylation may occur relatively rapidly, allowing the timing of specific regulatory events to be orchestrated more accurately. This is especially important during neurite formation, since other means of regulation, such as changes in gene expression, would be too slow to adequately regulate some aspects of this process. Since changes in morphology rely heavily on cytoskeletal rearrangements, the regulation of the cytoskeleton has clear importance in neurite formation. Thus, kinases known to regulate microtubule or actin dynamics have been the focus of much investigation.

However, kinases that have traditionally been thought about in different contexts are rapidly gaining interest in the neurite formation field. The Aurora and Nuak kinases are an excellent example of this. The Aurora kinases are well-characterized as mitotic kinases. Recently, Aurora Kinase A has been shown to play a critical role in neuronal migration in the developing cortex^12^. Additionally, Aurora Kinase A promotes neurite elongation in dorsal root ganglion cells and primary cortical neurons by reorganizing microtubules via an atypical protein kinase C (aPKC)-Aurora A-NUDEL1 pathway^13^. Furthermore, Aurora Kinase B overexpression results in extended axonal outgrowth, while pharmacological and genetic impairment of Aurora Kinase B activity caused truncation and aberrant motor axon morphology in zebrafish spinal motor neurons^14^. The role of Aurora Kinase C in neurite formation and neuromorphogenesis is currently unknown. Additionally, there are approximately 30 Aurora kinase inhibitors in either preclinical, phase I, or phase II clinical studies^15^. While many of these studies have been related to cancer, the clinical attention focused on Aurora kinase inhibitors makes them an interesting target for expanding their potential applicability, since they are on track to becoming pre-approved drugs.

Neurite formation is a highly energy-dependent process, but this aspect is overlooked when studying regulatory mechanisms of neuritogenesis. The Nuak kinases are AMPK-related kinases that have an established role in maintaining cellular homeostasis. Nuak Kinase 1 and Nuak Kinase 2 have roles in neural tube formation, with *NUAK1* and *NUAK2* double knockout mice exhibiting exencephaly, facial clefting, and spina bifida^16^. Nuak Kinase 1 is also involved in cortical axon arborization in vivo, with knockdown or overexpression of NUAK1 drastically reducing or increasing axon branching, respectively^17^. Mutations in *Nuak1* have been linked to autism spectrum disorder (ASD) and attention-deficit/hyperactivity disorder (ADHD)^18,19^. Since patients with these mutations are heterozygous carriers, a possible explanation for how *Nuak1* mutations exert their effect would be if *Nuak1* is haploinsufficient^20^. Thus, haploinsufficiency of *Nuak1* was investigated in mice and was shown to cause defects in the development of cortical connectivity and behavioral deficits such as spatial memory consolidation, social novelty, and abnormal sensorimotor gating^20^. With established roles in neurodevelopmental disorders, axon arborization, and cellular homeostasis, the Nuak kinases are a fascinating target of investigation. It is crucial to further investigate the Nuak kinases during all stages of neurodevelopment, including neurite formation.

We conducted a high-throughput screen of 493 kinase inhibitors using the DiscoveryProbe Kinase Inhibitor Library (APExBIO), which included inhibitors for both Ser/Thr and Tyr kinases, to identify kinases involved in neurite formation. We found that 45% of kinase inhibitors tested produced a neurite formation phenotype. The Aurora kinases A, B, and C and Nuak kinases 1 and 2 were implicated by our screening, prompting further investigation of their roles in neurite formation. Since the Aurora and Nuak kinases have well-established functions in regulating microtubule dynamics during mitosis and current literature indicates that these kinases may also be regulating similar functions during neuritogenesis, there is a need to further clarify the specific roles of the Aurora and Nuak kinases in neurite initiation and neurite elongation.

To further investigate the Aurora and Nuak kinases and confirm our screening we used a variety of pharmacological inhibitors to target individual kinases, as well as groups of kinases within each family. Our results suggest a crucial role for Aurora Kinase B and Nuak Kinase 1 in neuromorphogenesis, while inhibition of a combination of Aurora Kinase A/B, B/C, and A/B/C, as well as Nuak Kinase1/2 produced significant defects in neuronal morphology. Since our loss-of-function experiments using inhibitors indicate severe defects in neurite formation, we also tested the gain-of-function effects of each kinase on neurite formation. Overexpression of Aurora kinases A, B, and C in primary cortical neurons revealed that Aurora Kinase A is involved in neurite initiation, while Aurora kinases A, B, and C have roles in dendritic branching. Overexpression of Nuak kinases 1 and 2 in primary cortical neurons implicates Nuak Kinase 1 in neurite initiation and dendritic branching. These kinases could potentially serve as therapeutic targets for the treatment of central nervous system injury, seizures, and neurodegenerative disorders.

## Results

### Kinase inhibitor screening revealed that multiple kinases are involved in neurite formation

**Figure 1:**
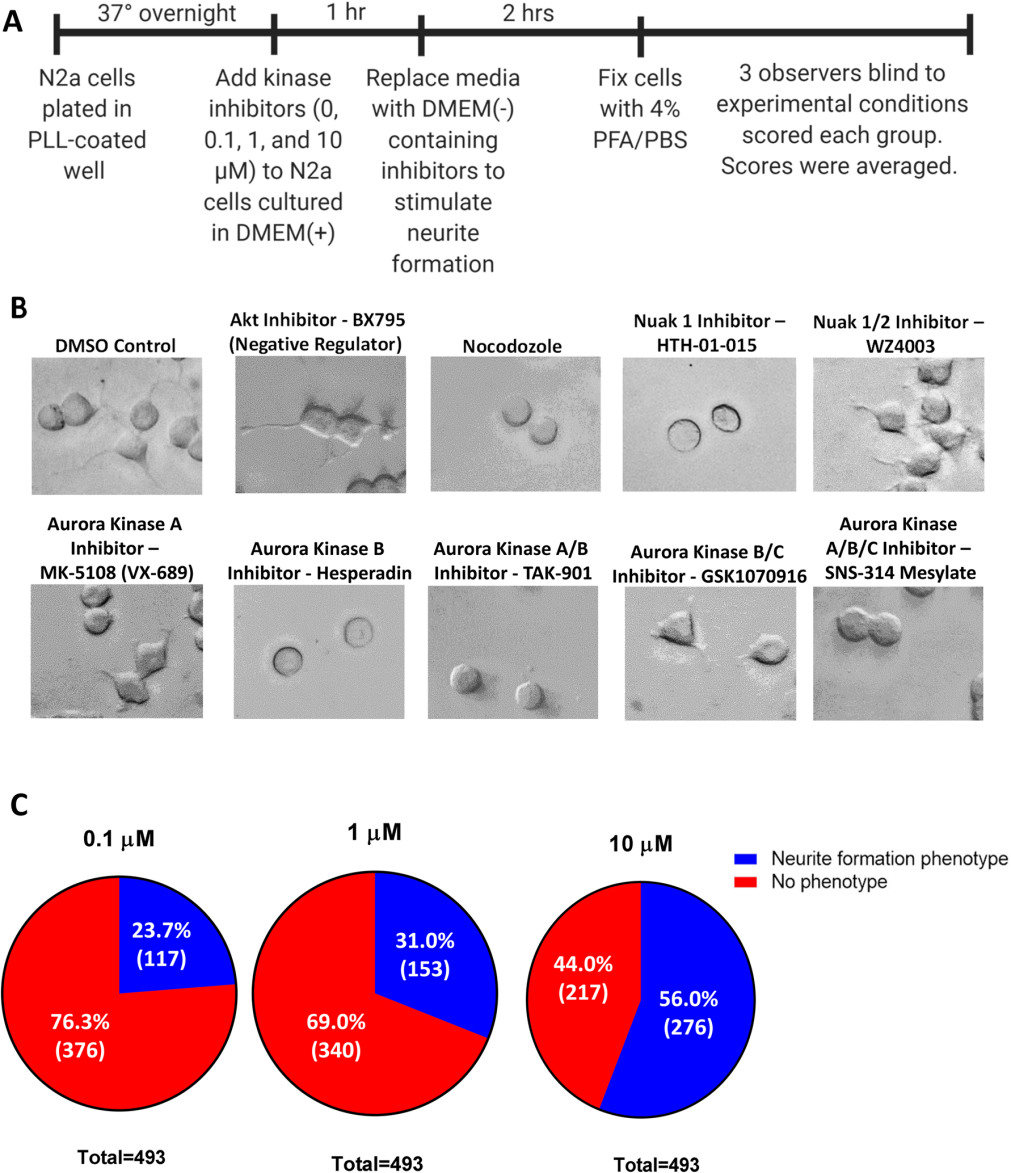

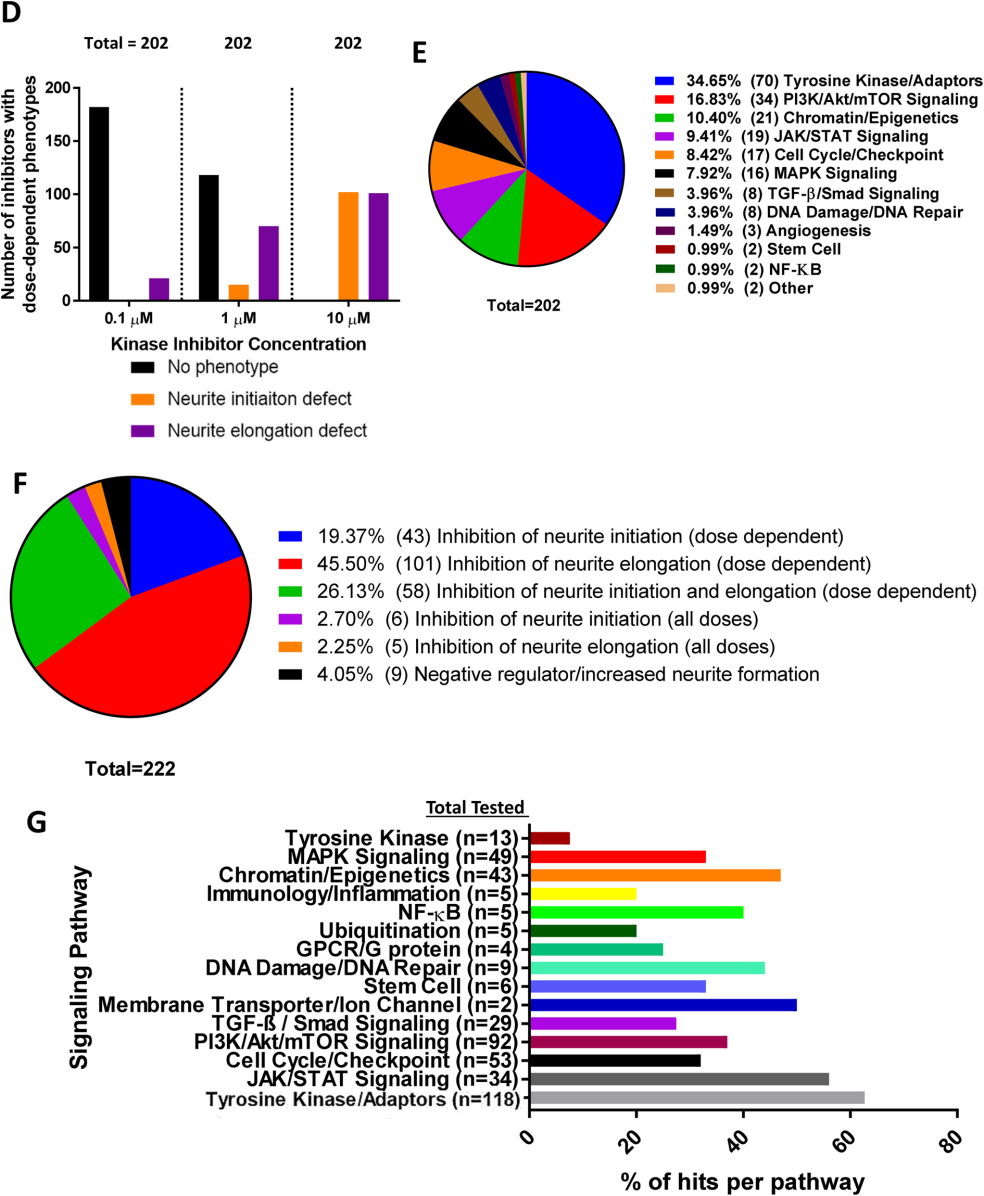
Kinase inhibitor screening revealed neurite formation phenotype for 222 kinase inhibitors. (**A**) Timeline describing the experimental design of the kinase inhibitor screening. (**B**) N-2a cells treated with 5 µM DMSO, Akt inhibitor—BX795, Nocodozole, Nuak 1 inhibitor—HTH-01–015, Nuak 1/2 inhibitor—WZ4003, Aurora Kinase A inhibitor—MK-5108 (VX-689), Aurora Kinase B inhibitor—Hesperadin, Aurora Kinase A/B inhibitor—TAK-901, Aurora Kinase B/C inhibitor—GSK1070916, Aurora Kinase A/B/C inhibitor—SNS-314 Mesylate. (**C**) Pie charts showing the percentage of groups that showed a neurite formation phenotype at each inhibitor dose. (**D**) Graph showing the number of dose-dependent inhibitors that had no phenotype, neurite initiation defect, or neurite elongation defect for each inhibitor dose group. (**E**) Pie chart showing the percentage of each signaling pathway that makes up the dose-dependent inhibitors group. (**F**) Summary of kinase inhibitor screening results shows the percentage of kinase inhibitors that resulted in each neurite formation phenotype. Percentages were taken out of the 45% of all inhibitors tested that had an effect on neurite formation, not the total number of inhibitors tested. (**G**) Summary of signaling pathways that kinases implicated in the regulation of neurite formation were most commonly found in, which displays the number of total kinase inhibitors tested on kinases in each signaling pathway and the percentage of those kinase inhibitors that produced a neurite formation phenotype for each. Data from dose-dependent inhibitors, inhibitors of negative regulators, and inhibitors with consistent phenotypes across all doses are represented.

To identify kinases that are involved in the regulation of neurite formation, we conducted a high-throughput screening by treating Neuro2a (N-2a) mouse neuroblastoma cells, which are induced to extend neurite-like extensions by FBS depletion from culture media, with kinase inhibitors (Fig. 1A). Briefly, N-2a cells were cultured for 1 h in DMEM (+) media containing kinase inhibitors. After 1 h, the media was changed to DMEM (−) with inhibitors to induce neurite outgrowth. Each of the 493 kinase inhibitors investigated were tested at concentrations of 0.1, 1, and 10 µM to optimize the dosage. Cells were fixed after 2 h. Three blinded observers used a scoring system to rank the extent of neurite formation in each condition by assigning each a score from 0 to 8 (Table 1). Scores were averaged and normalized to controls (see “Methods” for more detail). We observed that treatment with some inhibitors resulted in either no neurite formation or the formation of short neurites (Fig. 1B). We also observed that some inhibitors caused longer neurites compared to the control, suggesting that the kinases inhibited are negative regulators of neurite formation (Fig. 1B). Of the total 493 kinase inhibitors screened, 222 kinase inhibitors, or 45%, caused a neurite formation phenotype in N-2a cells for at least one of the three inhibitor concentrations tested. The percentage of kinase inhibitors that showed a neurite formation phenotype increased as inhibitor dose increased, and some inhibitors produced phenotypes at multiple or all doses (Fig. 1C). Inhibitors produced either no phenotype, a dose-dependent phenotype, or a consistent phenotype across all doses. Of the 202 kinase inhibitors that showed a dose-dependent phenotype that increased in severity as the dose increased, neurite elongation phenotypes were more common at lower doses (0.1 and 1 µM), while neurite initiation and neurite elongation phenotypes were about equally prevalent at the higher dose (10 µM) (Fig. 1D). Together the tyrosine kinase/adaptors and PI3K/Akt/mTOR signaling pathways accounted for about half of the total 203 kinases that showed a dose-dependent phenotype, with various other pathways accounting for the rest of the dose-dependent group (Fig. 1E). Of the 45% of kinase inhibitors that caused a neurite formation phenotype for at least one inhibitor concentration, 19.37% inhibited neurite initiation and 45.50% inhibited neurite elongation in a dose dependent manner, 26.13% inhibited both neurite elongation and initiation in a dose dependent manner, 2.70% inhibited neurite initiation at all doses, 2.25% inhibited neurite elongation at all doses, and 4.05% acted as negative regulators to increase neurite formation (Fig. 1F; Table 2). The screening also suggested prevalent roles in neurite formation for certain signaling pathways across all groups, including dose-dependent inhibitors, inhibitors of negative regulators, and inhibitors with consistent phenotypes at multiple doses, with the tyrosine kinase/adaptors, JAK/STAT, membrane transporter/ion channel, and chromatin/epigenetics signaling pathways being the top pathways shown to involve kinases that regulate neurite formation overall (Fig. 1G; Suppl. Table S1).

**Table 1:**
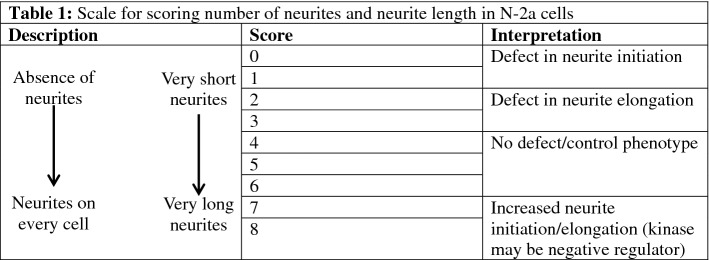
Scale used by three blind observers for scoring number of neurites from soma and neurite length in N-2a cells.

The average score for each condition was calculated and used to determine if each kinase inhibitor had an effect on the extent of neurite initiation or elongation.

**Table 2:** Kinase inhibitors that were determined to be negative regulators of neurite formation at 10 µM and regulators of neurite initiation or elongation at all doses (0.1, 1, 10 µM).

Kinase inhibitor	Pathway	Target kinase
**Negative regulators (10 µM)**		
SNS-032 (BMS-387032)	Cell cycle/checkpoint	Cyclin-dependent kinases
D4476	Stem cell	CK1
BX795	PI3K/Akt/mTOR signaling	Akt
R406	Tyrosine kinase/adaptors	Spleen tyrosine kinase (Syk)
GNF 5	TGF-β / Smad signaling	Bcr-Abl
CCT128930	PI3K/Akt/mTOR signaling	Akt
LEE011	Cell cycle/checkpoint	Cyclin-dependent kinases
Icotinib	JAK/STAT signaling	EGFR
NSC 23766	Cell cycle/checkpoint	Rho
**Regulators of neurite initiation (all doses)**		
WAY-600	PI3K/Akt/mTOR signaling	mTOR
TCS JNK 5a	MAPK signaling	JNK
KX2-391	Tyrosine kinase	Src
KX2-391 dihydrochloride	Tyrosine kinase/adaptors	Src
Tivantinib (ARQ 197)	Tyrosine kinase/adaptors	c-MET
Nocodazole	Ubiquitination	Autophagy
**Regulators of Neurite Elongation (all doses)**		
PP 1	Tyrosine kinase/adaptors	Src
ZCL278	Cell cycle/checkpoint	Cdc42
PH-797804	MAPK signaling	p38
ML347	TGF-β / smad signaling	TGF-βR1(ALK5)
Torin 2	PI3K/Akt/mTOR signaling	mTOR

The mean neurite formation scores were used to identify kinases involved in regulating the two stages of neurite formation: neurite initiation and neurite elongation. We considered the effect that kinase inhibitors had on both the number and length of neurites. No or primitive neurite formation reflects a defect in neurite initiation, while decreased neurite length is considered to be a defect in neurite elongation (Table 1). Based on the scores, we selected the kinases in which treatment with inhibitors targeting that kinase resulted in severe defects in neurite formation to investigate further. In some cases, multiple inhibitors in the library targeted the same kinase. The reported mean scores represent the average of all inhibitors that target each kinase.

Our kinase inhibitor screening in N-2a cells revealed neurite formation defects when the Aurora Kinases A, B, and/or C and Nuak Kinases 1 and/or 2 were inhibited at multiple doses (0.1, 1, 10 µM), suggesting a potential role for these kinases in neurite formation (Table 3). To inhibit Aurora kinases, we used kinase inhibitors targeting either Aurora Kinase A (MK-5108 (VX-689)), B (Hesperadin), A/B (TAK-901), B/C (GSK1070916), or A/B/C (SNS-314 Mesylate) (Fig. 1B). There were 25 kinase inhibitors that target Aurora kinases included in the inhibitor library. The mentioned kinase inhibitors were chosen to display representative images of the screening (Fig. 1B). MK-5108 (VX-689) is a novel, potent and selective inhibitor of Aurora A kinase that competitively binds to its ATP binding site^21^. Hesperadin is an ATP-competitive small molecule inhibitor of Aurora B kinase^22,23^. TAK-901 is derived from the novel azacarboline kinase hinge binder and has been shown to inhibit Aurora Kinase A and B^24^. GSK1070916 is a potent, selective, and reversible ATP-competitive inhibitor of Aurora Kinase B and C^25^. SNS-314 Mesylate is an ATP-competitive and selective inhibitor of Aurora kinase A, B, and C^26^. Inhibition of Aurora Kinase A resulted in neurite elongation defects, while inhibition of Aurora Kinase B resulted in neurite initiation defects. However, when both Aurora Kinase A and Aurora Kinase B were inhibited, neurite initiation defects were observed. Additionally, combined inhibition of Aurora Kinase B and Aurora Kinase C led to neurite elongation defects, while inhibition of all Aurora kinases A, B, and C caused neurite initiation defects. This suggests that the specific member or combination of members that are inhibited in the Aurora kinase family impacts the extent that neurite initiation and/or neurite elongation is affected. To inhibit Nuak kinases, we used kinase inhibitors targeting either Nuak Kinase 1 (HTH-01-015) or 1/2 (WZ4003). HTH-01-015 shows extreme selectivity for Nuak 1 and inhibits its phosphorylation, WZ4003 is a potent and selective inhibitor of Nuak kinases 1 and 2^27^. When Nuak Kinase 1 was inhibited, defects in neurite initiation were observed. Neurite elongation deficits were caused upon inhibition of both Nuak kinases 1 and 2. Thus, inhibition of both Nuak Kinase 1 and Nuak Kinase 2 results in a more severe phenotype.

**Table 3:** Mean neurite formation defect scores for kinase inhibitors targeting the Aurora and Nuak kinases reveal possible defects in neurite initiation and/or elongation.

Kinase inhibitor targets	Average score	Possible defect
DMSO control	5.04	N/A
Aurora Kinase A	3.69	Neurite elongation
Aurora Kinase B	1.22	Neurite initiation
Aurora Kinase A/B	1.18	Neurite initiation
Aurora Kinase B/C	3.74	Neurite elongation
Aurora Kinase A/B/C	1.46	Neurite initiation
Nuak 1	1.09	Neurite initiation
Nuak 1/2	3.43	Neurite elongation

### Inhibition of Aurora kinases in primary cortical neurons suggests roles for Aurora B and C in neurite initiation, elongation and arborization

**Figure 2:**
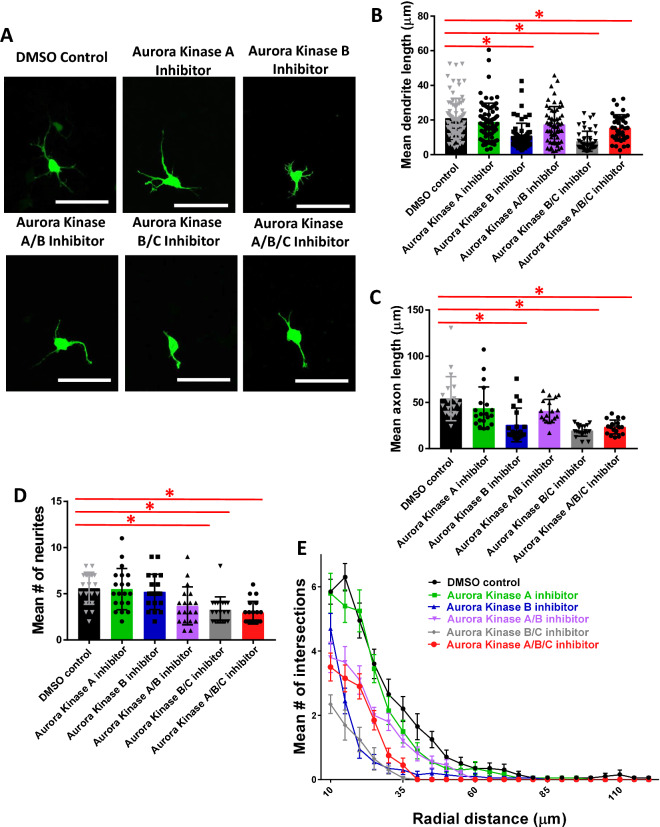
Inhibition of Aurora Kinases A, B, A/B, B/C, and A/B/C in primary cortical neurons suggests roles for Aurora B and C in neurite initiation/elongation and arborization. (**A**) Primary cortical mouse neurons transfected with YFP and treated with Aurora kinase inhibitors, scale bars 50 µm. (**B**) One-way ANOVA showed significant difference in length of neurites likely to become dendrites [F(5,354) = 16.941, *p* < 0.0005]. There was a significant decrease in neurons treated with Aurora B inhibitor (10.6 ± 0.95, n = 64, *p* < 0.0005), Aurora B/C inhibitor (7.7 ± 0.86, n = 46, *p* < 0.0005), or Aurora A/B/C inhibitor (15.5 ± 1.21, n = 39, *p* = 0.49) compared to DMSO control (21.0 ± 1.29, n = 80). There was no significant difference between neurons treated with Aurora A inhibitor (18.9 ± 1.26, n = 74, *p* = 1.000) or Aurora A/B inhibitor (17.4 ± 1.38, n = 57, *p* = 0.437) compared to DMSO control. (**C**) One-way ANOVA showed significant difference in length of neurites likely to become axons [F(5,111) = 12.946, *p* < 0.0005]. There was a significant decrease in neurons treated Aurora B inhibitor (25.7 ± 4.08, n = 20, *p* < 0.0005), Aurora B/C inhibitor (19.6 ± 1.36, n = 20, *p* < 0.0005), or Aurora A/B/C inhibitor (23.4 ± 1.73, n = 19, *p* < 0.0005) compared to DMSO treated control (54.1 ± 5.33, n = 20). There was no significant difference between neurons treated with Aurora A inhibitor (43.8 ± 5.13, n = 20, *p* = 0.860) or Aurora A/B inhibitor (40.7 ± 2.98, n = 18, *p* = 0.247) compared to DMSO control. (**D**) One-way ANOVA showed significant difference in mean number of neurites [F(5,113) = 8.941, *p* < 0.0005]. There was a significant decrease in neurons treated with Aurora A/B inhibitor (3.68 ± 0.47, n = 19, *p* = 0.017), Aurora B/C inhibitor (3.25 ± 0.32, *p* = 0.001), and Aurora A/B/C inhibitor (2.95 ± 0.28, *p* < 0.0005) compared to the DMSO treated controls (5.60 ± 0.39). There was no significant difference between neurons treated with Aurora A inhibitor (5.50 ± 0.50, *p* = 1.000) or Aurora B inhibitor (5.20 ± 0.43, *p* = 1.000) and DMSO control. For all groups, n = 20 unless otherwise noted. (**E**) Sholl analysis shows dramatic decrease in branching after treatment with Aurora Kinase B, A/B, B/C, and A/B/C inhibitors and a mild decrease in branching after Aurora Kinase A inhibitor treatment.

To further investigate the roles of the Aurora kinases and confirm the results of our N-2a cell screening, neurons expressing YFP were treated with kinase inhibitors targeting either Aurora Kinase A (MK-5108 (VX-689)), B (Hesperadin), A/B (TAK-901), B/C (GSK1070916), or A/B/C (SNS-314 Mesylate) (Fig. 2A). These Aurora kinase inhibitors were used in previous publications and the ability of the inhibitors to block downstream signaling is well validated^24,28–32^. Additionally, MK-5108, GSK1070916, and SNS-314 have already been used in one clinical trial each, indicating that safety profile for these inhibitors is already beginning to be established and providing strong rationale for selecting these inhibitors for use^29,33,34^. We considered the longest neurite the neurite most likely to become the axon, with the remaining neurites likely to become dendrites. Neurons treated with an Aurora Kinase B inhibitor (10.6 ± 0.95), Aurora Kinase B/C inhibitor (7.7 ± 0.86), or Aurora Kinase A/B/C inhibitor (15.5 ± 1.21) had significantly shorter neurites likely to become dendrites as compared to the DMSO treated control (21.0 ± 1.29) (Fig. 2B). Length of neurites likely to become axons was similarly affected, as neurons treated with an Aurora Kinase B inhibitor (25.7 ± 4.08), Aurora Kinase B/C inhibitor (19.6 ± 1.36), or Aurora Kinase A/B/C inhibitor (23.4 ± 1.73) had significantly shorter neurites likely to become axons as compared to the DMSO treated control (54.1 ± 5.33) (Fig. 2C). Additionally, the number of neurites was significantly decreased in neurons treated with an Aurora Kinase A/B inhibitor (3.68 ± 0.47), Aurora Kinase B/C inhibitor (3.25 ± 0.32), and Aurora Kinase A/B/C inhibitor (2.95 ± 0.28) as compared to the DMSO treated controls (5.60 ± 0.39) (Fig. 2D). While most experimental groups showed a notable decrease in arborization, neurons treated with the Aurora Kinase A inhibitor showed the mildest phenotype with a Sholl profile like DMSO treated control neurons (Fig. 2E). These data suggest that Aurora Kinase B and C are the major isoforms involved in multiple stages of neurite morphogenesis in primary cortical neurons.

### Inhibition of Nuak kinases 1 or 1/2 in primary cortical neurons suggests roles for Nuak 1 and 2 in neurite initiation and elongation and arborization

**Figure 3:**
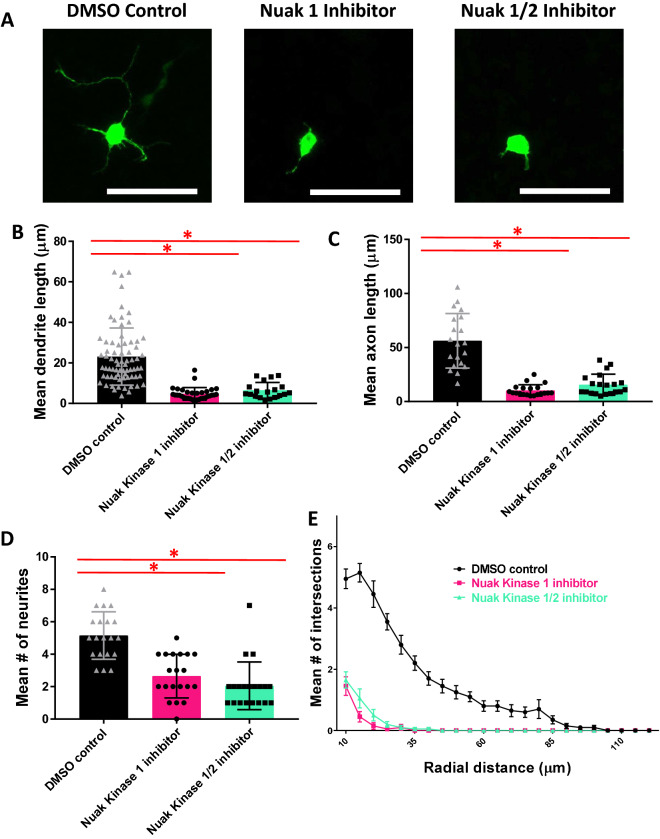
Inhibition of Nuak Kinases 1 and 1/2 in primary cortical neurons suggests roles for Nuak 1 and 2 in neurite initiation and elongation and arborization. (**A**) Primary cortical mouse neurons transfected with YFP and treated with Nuak kinase inhibitors, scale bars 50 µm. (**B**) One-way ANOVA determined there was a statistically significant difference in length of neurites likely to become dendrites [F(2, 120) = 39.341, *p* < 0.0005]. There was a statistically significant decrease in neurons treated with Nuak Kinase 1 inhibitor (4.7 ± 0.54, n = 33, *p* < 0.0005) and Nuak Kinase 1/2 inhibitor (6.48 ± 0.90, n = 19, *p* < 0.0005) as compared to the DMSO treated control (23.12 ± 1.67, n = 71). (**C**) One-way ANOVA determined there was a statistically significant difference in length of neurites likely to become axons [F(2, 57) = 49.832, *p* < 0.0005]. There was a statistically significant decrease in neurons treated with Nuak Kinase 1 inhibitor (10.3 ± 1.17, *p* < 0.0005) and Nuak Kinase 1/2 inhibitor (15.6 ± 2.19, *p* < 0.0005) as compared to the DMSO treated control (56.2 ± 5.64). For all groups, n = 20. (**D**) One-way ANOVA determined there was a statistically significant difference in mean number of neurites [F(2, 57) = 26.556, *p* < 0.0005]. There was a statistically significant decrease in neurons treated with Nuak Kinase 1 inhibitor (2.65 ± 0.30, *p* < 0.0005) and Nuak Kinase 1/2 inhibitor (2.05 ± 0.33, *p* < 0.0005) as compared to the DMSO treated control (5.15 ± 0.33). For all groups, n = 20. (**E**) Sholl analysis shows dramatic decrease in branching for neurons treated with Nuak Kinase 1 and 1/2 inhibitors.

To further investigate the roles of the Nuak kinases and confirm the results of our N-2a cell screening, neurons expressing YFP were treated with kinase inhibitors targeting either Nuak Kinase 1 (HTH-01-015) or 1/2 (WZ4003) (Fig. 3A). These Nuak kinase inhibitors were used in previous publications and the ability of the inhibitors to block downstream signaling is well validated^27^. Furthermore, TAK-901 has already been used in two phase I clinical trials, which was our rationale for selecting it for use in this study (clinicaltrials.gov). Neurons treated with a Nuak Kinase 1 inhibitor (4.7 ± 0.54) or Nuak Kinase 1/2 inhibitor (6.48 ± 0.90) had significantly shorter neurites likely to become dendrites as compared to the DMSO treated control (23.1 ± 1.67) (Fig. 3B). Length of neurites likely to be axons was similarly affected, as neurons treated with a Nuak Kinase 1 inhibitor (10.3 ± 1.17) or Nuak Kinase 1/2 inhibitor (15.6 ± 2.19) had significantly shorter neurites likely to be axons as compared to the DMSO treated control (56.2 ± 5.64) (Fig. 3C). Additionally, the number of neurites was significantly decreased in neurons treated with a Nuak Kinase 1 inhibitor (2.65 ± 0.30) or Nuak Kinase 1/2 inhibitor (2.05 ± 0.33) as compared to the DMSO treated control (5.15 ± 0.33) (Fig. 3D). Neurons treated with either the Nuak Kinase 1 or Nuak Kinase 1/2 inhibitor showed a dramatic decrease in arborization (Fig. 3E). These data strongly indicate the importance of the Nuak kinases in neurite morphogenesis in primary cortical neurons.

### Overexpression of Aurora Kinases A, B, or C in primary cortical neurons suggests role for Aurora Kinase A in neurite initiation and for Aurora Kinase A, B, and C in dendritic branching

**Figure 4:**
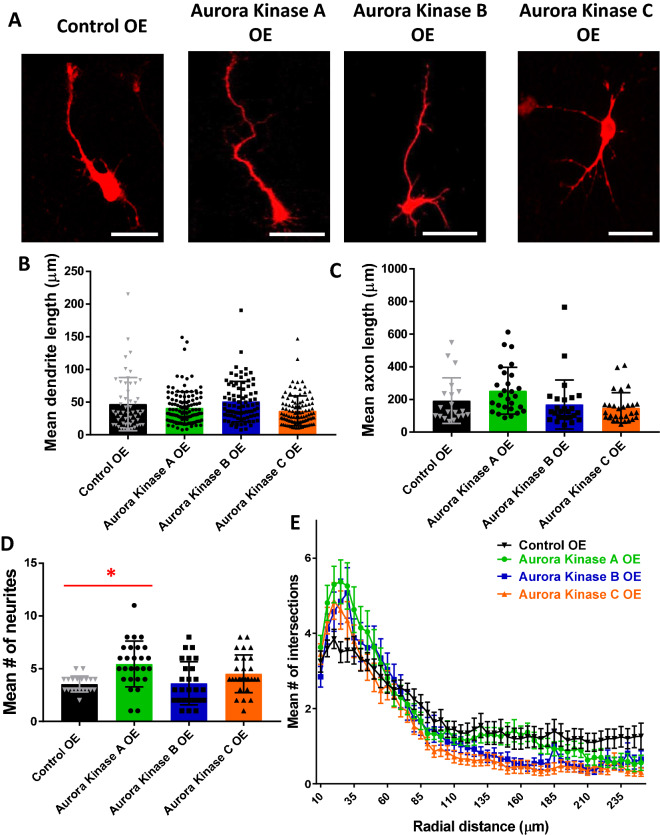
Overexpression of Aurora kinases A, B, and C in primary cortical neurons suggests role for Aurora Kinase A in neurite initiation and for Aurora Kinase A, B, and C in dendritic branching. (**A**) Primary cortical mouse neurons transfected with mRFP at 48 h after re-plating, scale bars 50 µm. (**B**) One-way ANOVA determined there was a statistically significant difference in length of neurites likely to become dendrites [F(3,354) = 4.256, *p* = 0.006]. However, there was no statistically significant difference between Aurora Kinase A OE (41.06 ± 2.28, n = 120, *p* = 1.000), Aurora Kinase B OE (50.84 ± 3.41, n = 82, *p* = 1.000), and Aurora Kinase C OE (36.56 ± 2.27, n = 102, *p* = 0.197) compared to control OE (46.92 ± 5.54, n = 54). (**C**) One-way ANOVA determined there was a statistically significant difference in length of neurites likely to become axons [F(3,98) = 3.209, *p* = 0.026]. However, there was no statistically significant difference between Aurora Kinase A OE (253.54 ± 27.58, n = 27, *p* = 0.745), Aurora Kinase B OE (169.50 ± 29.48, n = 26, *p* = 1.000), and Aurora Kinase C OE (149.26 ± 17.27, n = 29, *p* = 1.000) compared to control OE (193.05 ± 31.27, n = 20). (**D**) One-way ANOVA determined there was a statistically significant difference [F(3,98) = 5.991, *p* = 0.001]. Bonferroni post hoc test revealed that mean number of neurites was statistically significantly greater for Aurora Kinase A OE (5.4 ± 0.418, n = 27) compared to control OE (3.6 ± 0.170, n = 20) (*p* = 0.004). There was no statistically significant difference in mean number of neurites between Aurora Kinase B OE (3.6 ± 0.400, n = 26, *p* = 1.000) and Aurora Kinase C OE (4.5 ± 0.332, n = 29, *p* = 0.429) as compared to control OE (3.6 ± 0.170, n = 20). (**E**) Sholl analysis shows increase in branching complexity in Aurora Kinase A, B, and C overexpressing cells proximally, with a decrease in branching complexity distally as compared to control OE.

Since our experiments using inhibitors indicate that Aurora kinases are a positive regulator of multiple steps of neurite morphogenesis, we investigated the effects of overexpression of the Aurora kinases on neurite formation to assess the therapeutic potential of activating specific isoforms to increase neurite initiation and/or elongation. Plasmids overexpressing Aurora Kinases A, B, and C were validated via Western blot (See Supplemental Fig. S1). Mouse primary cortical neurons were used to overexpress the Aurora kinases A, B, or C (Fig. 4A). Neurons were re-plated, which re-starts the process of neurite formation and is a recognized model of neurite regeneration^35,36^. The length of neurites likely to become dendrites and neurites likely to become the axon, as well as number of neurites and arborization were analyzed, however no significant differences were found in the length of neurites likely to become dendrites and the axon (Fig. 4B,C). Interestingly, neurons overexpressing Aurora Kinase A (5.4 ± 0.418) had significantly more neurites as compared to controls (3.6 ± 0.170) (Fig. 4D). The Aurora kinases A, B, and C all showed increased dendritic branching proximally and decreased dendritic branching distally (Fig. 4E). These data suggest that Aurora Kinase A is involved in neurite initiation and that all Aurora kinases are involved in dendritic branching, but have different roles in proximal and distal regions of dendrites. Additionally, overexpression of Aurora Kinase A may have therapeutic applications following central nervous system injury to induce axon regeneration.

### Overexpression of Nuak Kinases 1 or 2 in primary cortical neurons suggests roles for Nuak Kinase 1 in neurite initiation and dendritic branching

**Figure 5:**
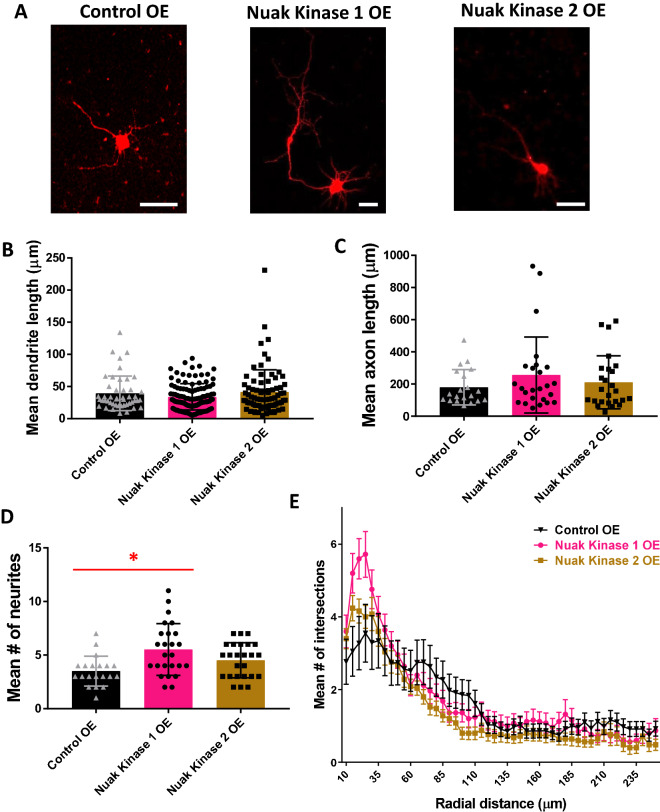
Overexpression of Nuak kinases 1 and 2 in primary cortical neurons suggests roles for Nuak Kinase 1 in neurite initiation and dendritic branching. (**A**) Primary cortical mouse neurons transfected with mRFP at 48 h after re-plating, scale bars 50 µm. (**B**) One-way ANOVA determined there was no statistically significant difference in length of neurites likely to become dendrites [F(2,234) = 1.904, *p* = 0.151]. There was no statistically significant difference between Nuak Kinase 1 OE (34.06 ± 1.94, n = 111, *p* = 0.742) and Nuak Kinase 2 OE (41.56 ± 3.89, n = 79, *p* = 1.000) as compared to control OE (39.53 ± 3.92, n = 47). (**C**) One-way ANOVA determined there was no statistically significant difference in length of neurites likely to become axons [F(2,67) = 1.010, *p* = 0.370]. There was no statistically significant difference between Nuak Kinase 1 OE (255.91 ± 47.27, n = 33, *p* = 0.500) and Nuak Kinase 2 OE (210.43 ± 33.01, n = 25, *p* = 1.000) as compared to control OE (179.47 ± 24.55, n = 20). (**D**) One-way ANOVA determined there was a statistically significant difference [F(2,67) = 6.313, *p* = 0.003]. Bonferroni post hoc test revealed that mean number of neurites was statistically significantly greater for Nuak Kinase 1 OE (5.5 ± 0.483, n = 25, *p* = 0.002) as compared to control OE (3.5 ± 0.312, n = 20). There was no statistically significant difference in mean number of neurites between Nuak Kinase 2 OE (4.5 ± 0.327, n = 25, *p* = 0.233) as compared to control OE (3.5 ± 0.312, n = 20). (**E**) Sholl analysis shows a dramatic increase in branching complexity in Nuak Kinase 1 overexpressing cells proximally and a slight increase in branching complexity in Nuak Kinase 2 overexpressing cells proximally, with a decrease in branching complexity more distally in both Nuak kinases as compared to control OE. Branching appears to become similar to controls in both experimental groups at the most distal points.

To further investigate the role of the Nuak kinase isoforms in neurite formation, we validated plasmids overexpressing Nuak kinases 1 and 2 via Western blot (Suppl. Figs. S1, S2). Mouse primary cortical neurons were then used to overexpress Nuak kinases 1 or 2. Following overexpression, neurons were re-plated to re-start the process of neurite formation, serving as a model of neurite regeneration^35,36^. Neuronal morphology was assessed to analyze the length of neurites likely to become dendrites and neurites likely to become the axon, number of neurites, and arborization (Fig. 5A). Length of neurites likely to become dendrites and neurites likely to become axons was assessed, but no significant differences were found (Fig. 5B,C). Neurons overexpressing Nuak Kinase 1 (5.5 ± 0.483) had significantly more neurites as compared to the control (3.5 ± 0.312, n = 20) (Fig. 5D). Branching was also dramatically increased proximally in neurons overexpressing Nuak Kinase 1 and slightly increased proximally in neurons overexpressing Nuak Kinase 2 (Fig. 5E). Additionally, overexpression of both Nuak kinases resulted in a slight decrease in arborization more distally, with branching that was similar to controls at the most extremely distal lengths (Fig. 5E). Another interesting aspect of morphology is the turning of the neurite likely to be the axon back towards the cell body and axonal swellings in neurons overexpressing Nuak Kinase 1 (Fig. 5A, middle panel). Thus, these data suggest that Nuak Kinase 1 is involved in neurite initiation and both Nuak kinases 1 and 2 are involved in dendritic branching. Further investigation is warranted to determine whether overexpression of Nuak Kinase 1 may be beneficial following traumatic injuries such as spinal cord injury, due to its involvement in neurite initiation and potential to induce axonal regeneration.

### Expression level and pattern changes of Aurora and Nuak kinases in primary cortical neurons prepared from ASD mouse model suggest role producing in neurite formation defects

**Figure 6:**
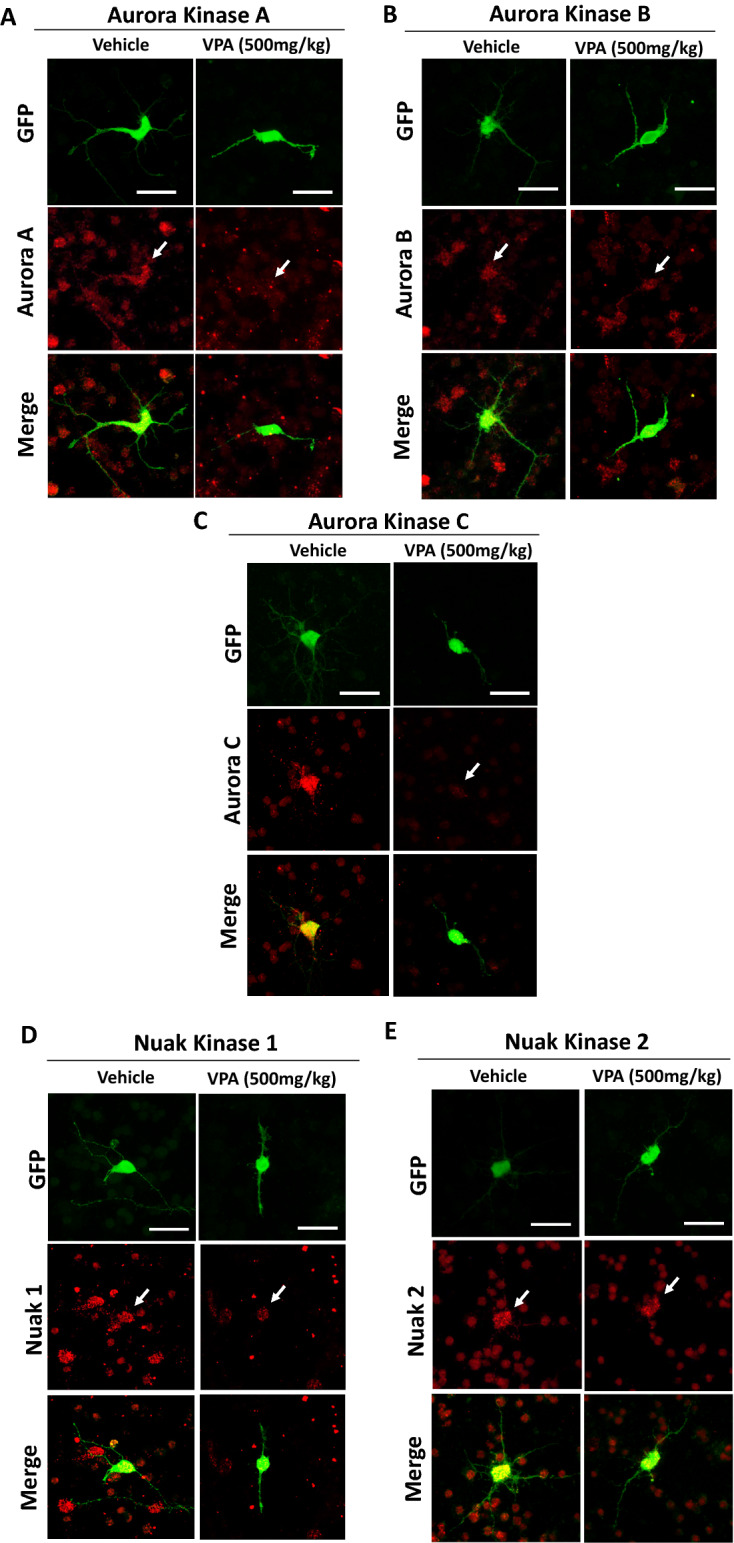
Expression level and pattern changes of Aurora and Nuak kinases in primary cortical neurons prepared from ASD mouse model suggest role producing in neurite formation defects. Primary cortical mouse neurons prepared from either vehicle or VPA injected dams were transfected with YFP and stained for Aurora Kinase A (**A**), Aurora Kinase B (**B**), Aurora Kinase C (**C**), Nuak Kinase 1 (**D**), and Nuak Kinase 2 (**E**), scale bars 20 µm. Note that Aurora and Nuak kinases expressed in the soma and the proximal region of neurites in the control neurons. In VPA-treated neurons, the expression of Aurora Kinase A and C as well as Nuak Kinase 1 was decreased in the soma and neurites.

It is well known that many animal models and human induced pluripotent stem cells (hiPSCs) generated from autism spectrum disorder (ASD) patients show neurite formation defects^37,38^. The treatment of pregnant dams with valproic acid (VPA) has been used as an idiopathic ASD model and induces cellular pathophysiology like that seen in ASD^37,38^. To understand whether the expression patterns of Aurora and Nuak kinases are affected in disease models in which neurite formation is deficient, we tested whether the expression patterns of Aurora and Nuak kinases are affected in VPA-treated neurons. Primary cortical neurons were prepared using E14.5 embryos from pregnant dams which were injected with either PBS or VPA at E9.5. Immunocytochemistry was performed on healthy neurons prepared from the PBS vehicle injection group and on neurons with various neuromorphological defects prepared from the VPA injection group. We found that Aurora Kinase A is highly expressed in the soma of healthy neurons and the proximal region of neurites with punctate staining pattern as previously reported^39^, while Aurora Kinase A expression dramatically decreases in the soma of VPA treated neurons but the strong spot-like staining at the somal edge region remains. (Fig. 6A). Aurora Kinase B is strongly expressed in the soma and neurites in control neurons with punctate staining pattern as previously reported^14,39^, while Aurora Kinase B expression in VPA treated neurons decreased slightly in the soma but was still moderately expressed in neurites (Fig. 6B). Aurora Kinase C is predominantly expressed in the soma of control neurons with low expression in the neurites, as compared to the VPA group which shows Aurora Kinase C expression is dramatically decreased in the soma and is not present in neurites (Fig. 6C). Nuak Kinase 1 is expressed in the soma in control neurons but appears to have lower expression in the nucleus and neurites, while prenatal exposure to VPA decreased Nuak Kinase 1 expression throughout the entire soma and neurites (Fig. 6D). Nuak Kinase 2 was strongly expressed in the soma and weakly in neurites in control. VPA exposed neurons showed a slightly decreased expression in the soma and no expression in neurites (Fig. 6E). These results suggest the potential involvement of Aurora Kinase A and C as well as Nuak Kinase 1 in neurite formation defects caused by prenatal exposure to VPA.

## Discussion

Our study reports a high-throughput kinase inhibitor screening, which implicates 222 kinase inhibitors as potential therapeutics. There is huge therapeutic potential for manipulation of kinase activity, as nearly 50 kinase inhibitors have already been approved by the FDA for use^10^. Most kinases that were targets of these inhibitors were members of the tyrosine kinase/adaptors, JAK/STAT, membrane transporter/ion channel, and chromatin/epigenetics signaling pathways, highlighting the importance of these pathways in neuritogenesis. We also report roles for Aurora Kinase A in neurite initiation, for Aurora kinases A, B, and C in dendritic branching, and for all Nuak kinases in neurite initiation and elongation and dendritic branching. With phase I clinical trials already completed on 4 of the 7 Aurora or Nuak kinase inhibitors used in our primary neuron experiments, drug development time for these inhibitors should be considerably decreased as compared to most other new drug candidates.

Our screening indicated a role for several pathways that are not traditionally thought to have a role in neurite formation including the tyrosine kinase/adaptors, JAK/STAT, membrane transporter/ion channel, and chromatin/epigenetics signaling pathways pathway in neuritogenesis. Several tyrosine kinases such as anaplastic lymphoma kinase (ALK) have known roles in neurite outgrowth and activated Cdc42-associated tyrosine kinase (ACK1) plays a role in neurite extension and branching^40,41^. The JAK/STAT pathway is typically thought of as negatively regulating neurite formation and acts through inhibitory proteins such as SOCS2, 3, and 6^42–44^. Kinases in the membrane transporter/ion channel pathways may regulate ion channels that are important for neurite formation. For example, calcium signaling is known to be an important regulator of neuritogenesis, since the rate of neurite extension is regulated by the frequency of spontaneous calcium transients^45–47^. Kinases in chromatin/epigenetics signaling pathways serve to regulate neurite formation through chromatin remodeling or post-translational modifications of transcription factors^48–50^. Other kinases in these pathways should be investigated more closely to determine if additional regulators of neurite formation may be identified.

Depending on the specific combination of Aurora and Nuak kinase isoforms inhibited in N-2a cells, defects in either neurite initiation or neurite elongation were observed. Thus, defining the specific function of each isoform proves challenging because in certain situations inhibition of a particular kinase may result in neurite initiation defects, while in a different situation when this kinase is inhibited in combination with different isoforms it may result in neurite elongation defects. Our results from the N-2a cell screening suggest that the specific combination of kinase isoforms inhibited within each family impacts the neurite morphology phenotype seen (Table 3). Potentially, inhibiting different combinations of Aurora kinases A, B, and/or C or Nuak kinases 1 and 2 could cause different compensatory pathways to be activated within the cells.

To expand upon our findings from the N-2a cell screening, we used primary cortical neurons and pharmacologically inhibited several individual Aurora and Nuak kinases, as well as multiple different combinations of isoforms within each family. Interestingly, inhibition of Aurora Kinase A, which was the only Aurora kinase to show an overexpression phenotype for number of neurites, resulted in the least severe inhibition phenotype, with no significant difference seen in neurite initiation or elongation and only a mild defect in arborization (Table 4). To inhibit Aurora Kinase A we used MK-5108, which is one of the most selective Aurora A inhibitors and determination of the Aurora A/MK-5108 crystal structure suggests a chemical basis for its high specificity^51^. MK-5108 has an IC_50_ of 0.064 nM, as determined by a biochemical in vitro assay without cells^52^. A study on the effects of MK-5108 in 14 different cell lines showed that the IC_50_ for MK-5108 in vivo ranges from 0.16 and 6.4 µM^52^. Since the actual optimal concentration depends on multiple factors, such as cell type and membrane permeability of the inhibitor, we determined the optimum dosage of 5 µM MK-5108 to maintain cell health in primary cortical neurons. This dose is consistent with previous studies^21,51^. While MK-5108 is a highly selective Aurora Kinase A inhibitor, it can also inhibit Aurora kinases B and C at much higher concentrations. The IC_50_ for both Aurora Kinase B and C is approximately 9 nM, as compared to the IC_50_ for Aurora Kinase A of 0.064 nM. We believe we selectively inhibited Aurora Kinase A with MK-5108 treatment, as we would have observed cell toxicity and death at a concentration high enough to inhibit Aurora Kinase B or C since we carefully optimized MK-5108 concentration for cell health (unpublished data)^52^.

**Table 4:** Summary table of the phenotypes observed in primary cortical neurons when each Aurora and Nuak kinase was either overexpressed or inhibited.

	Length of neurites likely to become dendrites (elongation)	Length of Neurites likely to become axons (elongation)	Neurite initiation	Arborization
Aurora A overexpression	X	X	Increased	Increased proximally, decreased distally
Aurora A inhibition	X	X	X	Mild decrease
Aurora B overexpression	X	X	X	Increased proximally, decreased distally
Aurora B inhibition	Decreased	Decreased	X	Decreased
Aurora A/B inhibition	X	X	Decreased	Decreased
Aurora B/C inhibition	Decreased	Decreased	Decreased	Decreased
Aurora A/B/C inhibition	Decreased	Decreased	Decreased	Decreased
Aurora C overexpression	X	X	X	Increased proximally, decreased distally
Nuak 1 overexpression	X	X	Increased	Increased proximally, decreased distally
Nuak 1 inhibition	Decreased	Decreased	Decreased	Decreased
Nuak 2 overexpression	X	X	X	Increased proximally, decreased distally
Nuak 1/2 inhibition	Decreased	Decreased	Decreased	Decreased

X = no significant difference.

Additional findings from our cortical neuron studies revealed that combined inhibition of Aurora Kinase A/B may only impact neurite initiation and arborization (Table 4). Inhibition of only Aurora Kinase B may specifically affect neurite elongation and arborization, while combined inhibition of Aurora Kinase B/C has a more severe phenotype compared to inhibition of Aurora Kinase A alone (Table 4). These results suggest that Aurora Kinase B plays a crucial role in regulating neuromorphogenesis, and that Aurora Kinase C also has functions in neuromorphogenesis. Inhibition of either Nuak Kinase 1 or Nuak Kinase 1/2 resulted in severe defects in neurite initiation, neurite elongation, and arborization (Table 4). However, we did not see any significant differences between neurons treated with the Nuak Kinase 1 inhibitor as compared with the Nuak Kinase 1/2 inhibitor. This suggests that Nuak Kinase 1 and 2 may have redundant functions or no functions during neuromorphogenesis, since inhibition of Nuak Kinase 2 in addition to Nuak Kinase 1 does not appear to have any additive effects.

These results highlight the crucial need to distinguish effects of kinase targeting drugs on different kinase isoforms and in different cell types. Attention must be paid to isoform specificity and how targeting different combinations of isoforms may lead to redundant or divergent effects. We used the N-2a cell screening as a high-throughput method to identify the Aurora and Nuak kinases as targets for investigation, since all isoforms showed a phenotype in N-2a cells. We then used the primary cortical neurons to study neurite formation in more detail and parse out whether each kinase was involved in neurite initiation, elongation of the neurite likely to become the axon, elongation of neurites likely to become dendrites, and/or arborization. Differences in results between N-2a cells may be due to cell-type specificity, sensitivity of the screenings, or method used to induce extension of neurites (if necessary). For example, our results from N-2a cells implicated Aurora Kinase A in neurite elongation, while our results in primary cortical neurons show no significant difference in length for neurites likely to become dendrites. This result is not particularly surprising, as kinases often have cell-type specific roles during neurite formation. Previous studies using different cell types, including hippocampal, cortical, and dopaminergic neurons, PC12 cells, and N-2a cells, have shown that Akt, AMPK, CaMK II, JNK, MAPK/ERK, and PKA all have cell-type specific roles^7,53–66^. Some kinases, like JNK, are even known to have pathway specific effects, while others such as AMPK may have different roles depending on subunit composition^67–75^. These differences are important to recognize and understand when designing targeted therapeutics, and differences in phenotypes between cell types should not be discounted.

The known roles of Aurora kinases A, B, and C during neurite formation, axon regeneration, and mitosis are similar yet distinct. Aurora Kinase A has a known role in neurite elongation, and our overexpression results further implicate Aurora Kinase A in neurite initiation^13^. Mori et al. found that depletion of Aurora Kinase A and any components of the signaling pathway resulted in decreased frequency and speed of microtubule emanation from the microtubule organizing center and into primitive neurites, and subsequent decreased neurite elongation^13^. Microtubule invasion into primitive neurites during neurite initiation is also crucial for neurite stabilization, dictating which lamellipodia and filopodia remain to undergo neurite elongation or retract. Because Mori et al. performed neurite analysis 48 h after transfection without re-plating, they likely were only able to observe elongation deficits^13^. Neurite initiation begins immediately upon plating before the transfection has taken full effect, and this experimental timing difference explains the further deficits noted in our Aurora Kinase A overexpression experiments which analyzed neurite initiation and elongation 48 h after re-plating after the transfection has reached full efficiency. Elongation results from Aurora Kinase A inhibition in primary cortical neurons is in agreement with previous literature in dorsal root ganglion cells, although we show a trend towards decreased neurite length that does not reach significance. We observed increased neurite initiation but did not observe changes to neurite elongation upon Aurora Kinase A overexpression. Overexpression is not a measure of protein function but can be useful to test the effects of manipulation of certain targets. This suggests that although Aurora Kinase A has important developmental roles in neurite elongation, its activation may not be useful to therapeutically target axon elongation of a damaged axon. Instead, it could be applied to axon re-initiation in cases where the axon must re-emerge from the soma.

Aurora Kinase B has a known role in axon outgrowth and regeneration in zebrafish^14^. Gwee et al. found that pharmacological inhibition of Aurora Kinase B prevented both axonal outgrowth and regeneration in spinal motor neurons, and that overexpression of Aurora Kinase B led to increased axon elongation^14^. Our kinase inhibitor results are in agreement with these results, and also revealed further deficits that were not specific to the axon but to all neurites. We found that Aurora Kinase B inhibition results in decreased dendrite and axon length in mouse primary cortical neurons. It is possible that cell and species type specific activity of Aurora Kinase B contributed to our additional results. Taken together, our study and others have provided strong evidence that Aurora Kinase B contributes to neurite elongation in post-mitotic cells. Surprisingly, overexpression of Aurora Kinase B had no significant effects on cortical neuron morphology. Aurora Kinase B’s canonical role in mitosis maintains chromosome alignment and segregation fidelity^76^. One of the many ways Aurora Kinase B promotes these mechanisms is through phosphorylation and activation of microtubule depolymerizer, Kif2A, which was recently shown to contribute to neuronal morphology^77^. It has been shown that in cases where Aurora Kinase B is altered during development, endogenous activity of Aurora Kinase C can rescue these deficits^78^. It is not unlikely that a compensatory mechanism of this nature could also take place during overexpression of either kinase.

Aurora Kinase C has overlapping canonical roles with Aurora Kinase B^76^. We are the first to analyze the role of Aurora Kinase C in post-mitotic cells. We discovered that Aurora Kinase C contributes to dendritic branching, implicating its inhibition as a therapeutically valuable target to trim back aberrant neurite branching following seizures. Because none of our kinase inhibitors were selective for only Aurora Kinase C, it is hard to interpret whether Aurora Kinase C is important for neurite formation as the results seen in our inhibitor assay could be due to combined inhibition of Aurora Kinase B. Since Aurora B and C have similar roles during mitosis, with one of their major functions being to organize microtubules, we may speculate that Aurora B and C may also function similarly to each other during neurite formation. This may explain why combined inhibition of Aurora B and C leads to defects. Additionally, Aurora A functions differently during mitosis, with one of its main roles being to regulate microtubule assembly and polymerization. This could explain why we only see an overexpression phenotype when Aurora A is overexpressed, since increased microtubule polymerization could increase neurite outgrowth, while increasing microtubule organizing regulators, which may be occurring when Aurora B and C are overexpressed, may not impact neurite formation^79^. Furthermore, Aurora B and C have known compensatory roles for each other when one is disrupted.

Our results suggest differential roles for kinases in the Aurora family. This information could be useful in designing therapeutics if the kinase structural domains that are relevant for affecting specific aspects of neuronal morphology can be identified. While the Aurora kinases are structurally similar, there are notable differences. Aurora Kinase B and Aurora Kinase C share 75% homology of entire sequence and kinase domain, while Aurora Kinase A and Aurora Kinase C share 60% homology of total sequence and kinase domain^80^. Future studies may identify which domains are involved in the regulation of axon and dendrite length by mutating several regions of each kinase that have unique homology to determine what effect each domain has on regulating neuromorphogenesis.

Kinase inhibitor therapeutics have the potential to be useful in a broad range of treatments targeting axon/dendrite degeneration or regeneration. For example, aberrant neurite formation may occur following seizure, resulting in the formation of inappropriate synaptic contacts^81^. Neuronal defects are also seen in neurodevelopmental disorders, with alterations in dendritic branching seen in patients with Rett Syndrome and Fragile-X^82^. Additionally, several neuromorphological deficits have been associated with neurodegenerative conditions. Increased numbers of primary neurites per neuron has been linked to expression of alpha-synuclein, which is a protein commonly associated with Parkinson’s disease^83^. Primary neuronal cultures expressing a version of amyloid-β protein called Aβ42, which is involved in Alzheimer’s disease, displayed an enhanced neurite outgrowth and arborization before neurodegeneration occurred^84^. A similar phenomenon was observed in motor neurons cultured from SOD1 amyotrophic lateral sclerosis (ALS) mice^85^. To improve clinical phenotypes, a targeted therapeutic may be designed to inhibit neurite initiation, elongation, and/or branching in these and other similar conditions where neurite formation and arborization are enhanced. Our results indicate that inhibition of Aurora and Nuak kinases can decrease neurite formation and arborization, and since several Aurora and Nuak kinase inhibitors are in clinical trials, they are strong potential candidates for such a therapeutic.

Although the overexpression of the kinases of interest is not a direct functional analysis, results from overexpression can suggest the functions of the protein and/or the utility of activating or overexpressing that protein. Our overexpression experiments indicate Aurora Kinase A and Nuak Kinase 1 are critical factors during neurite initiation and dendritic branching. The kinase inhibition and overexpression phenotypes did not match, which was expected. The purpose of the overexpression experiments was to assess the therapeutic potential of activating specific isoforms to increase neurite formation. Thus, the expected result was either no phenotype or an increase in neurite initiation and/or elongation. Furthermore, previous studies have reported that the knockdown phenotype, which is likely similar to an inhibitor phenotype, can be opposite or not match with overexpression results^86–89^. The overexpression experiments also tested whether overexpression of the Aurora and Nuak kinases have therapeutic benefit during neurite regeneration. Re-plating the neurons caused them to re-start neurite initiation, thus regenerating^35,36^. Our results indicate that overexpression of Aurora Kinase A and Nuak Kinase 1 increase neurite initiation in a neurite regeneration model. Increased activity of these kinases may induce axon outgrowth following traumatic injury by creating a more permissive intrinsic signaling environment within mature neurons, which typically fail to regenerate in the central nervous system for multiple reasons including poor intrinsic growth potential. Future studies should be done to advance our observations by performing scratch-based in vitro neurite regeneration assays in different types of neurons, as well as in vivo assays using animal models of brain injury and spinal cord injury.

Previous literature first reported a role for the Aurora kinases A and B and Nuak 1. Our overexpression phenotypes confirm these results as well as implicate the Aurora and Nuak kinases in additional aspects of neuromorphogenesis. Aurora Kinase A has a known role in neurite elongation and Aurora Kinase B has a known role in regulating axon length^12–14^. We show here that Aurora Kinase A is also involved in neurite initiation and that the Aurora kinases A, B, and C are all involved in dendritic branching. Previously, Nuak Kinase 1 has been reported to regulate axonal branching^17^. We describe roles for Nuak Kinase 1 in neurite initiation and dendritic branching as well. Our results further emphasize the importance of the Aurora and Nuak kinases in multiple steps of neurite formation and dendritic branching.

Traditional approaches have favored knockdown experiments. However, our goal was to investigate the potential applications for kinase inhibitors. MK-5108 (Aurora A inhibitor), GSK1070916 (Aurora B/C inhibitor), and SNS-314 (Aurora A/B/C inhibitor) have all been used in one clinical trial each already, while TAK-901 (Aurora A/B inhibitor) has already been used in two clinical trials. This indicates that the safety profile for these inhibitors is already beginning to be established and should expedite drug development time, providing a compelling reason to investigate the potential use of these kinase inhibitors. While using a knockdown approach would undoubtedly allow us to confirm whether the knockdown phenotype matches the inhibitor phenotype, this would not advance the goal of investigating kinase inhibitors in treating neuronal deficits.

The staining of Aurora and Nuak kinases in primary cortical neurons showed each kinase predominantly expressed in the soma and proximal region of neurites. The previous studies, which mainly used non-neuronal cells, indicate that Aurora and Nuak kinases express in the nucleus and cytoplasm. Interestingly, Aurora Kinase A expresses in the soma and centrosome in dorsal root ganglion neurons and cortical neurons and regulates neurite elongation and neuronal migration^12,13^. Therefore, it is possible that other Aurora kinases could regulate neurite elongation as well as arborization via regulating microtubule organization at the centrosome. Our cell staining experiment using VPA-treated neurons revealed that Aurora Kinase A expression was decreased in the soma, but the strong dot-like staining in the somal edge region was observed in VPA-treated neurons (Fig. 6A). This suggest that Aurora Kinase A functions in the soma, as well as the nucleus, are disrupted in VPA-treated neurons, and this causes defects in neurite elongation as seen in VPA-treated neurons (Fig. 6A). Nuak Kinase 1 expression was decreased in VPA-treated neurons, which is consistent with the previous study using Nuak Kinase 1 deficient mice in which Nuak Kinase 1 deficiency resulted in neurite formation defects (Fig. 6D)^20^. Thus, although it is known that Nuak Kinase 1 is a potential responsible gene of ASD, Aurora kinases could be implicated in ASD and other neurodevelopmental disorders in which neurite formation is disrupted.

In conclusion, our results support the previous observations reported from other groups, and novel roles for the Aurora and Nuak kinases in regulating neurite initiation and dendritic branching have been clarified. Inhibition and overexpression phenotypes also reveal differences within these kinase families, suggesting that each class of kinase plays a very specific role in establishing axons and dendrites. Additionally, the high-throughput kinase inhibitor screening we conducted has the potential to call attention to new targets of investigation in the search for better therapeutics to treat patients suffering from aberrant axon sprouting or failure to regenerate injured axons.

## Methods

### Mice

All animal experiments were performed under protocols approved by the Drexel University Animal Care and Use Committees and following the guidelines provided by the US National Institutes of Health. This study was carried out in compliance with the ARRIVE guidelines. C57BL/6 mice were maintained in our animal facility and used for setting up mating to obtain the time-pregnant mice. Embryonic day (E) 0.5 was defined as the morning of the day the vaginal plug appeared.

### Kinase inhibitor library

DiscoveryProbe kinase inhibitor library (Cat # L1024) was purchased from APExBIO (Boston, MA). The library is a collection of 493 kinase inhibitors, mainly including inhibitors, but also some activators. Original 10 mM in DMSO stocks were stored at − 80 °C, and 1 mM working solutions were created by diluting the stock solution in DMSO and also storing at − 80 °C until use. The full list of inhibitors is supplied as a supplemental table (Suppl. Table S2).

### Kinase inhibitor screening

Neuro-2a (N-2a) mouse neuroblastoma cells were plated in a 96 well plate that was coated with poly-l-lysine (PLL). N-2a cells were cultured for 1 h in DMEM (+) media containing kinase inhibitors from the DiscoveryProbe Kinase Inhibitor Library (APExBIO). After 1 h, the media was changed to DMEM (−) with inhibitors to induce neurite outgrowth. Each of the 493 kinase inhibitors investigated were tested at concentrations of 0.1, 1, and 10 µM to optimize the dosage, since there was a range of doses at which the kinase inhibitors could be administered before a toxic level was reached and cell death was observed. After 3 h cells were fixed. Three blinded observers used a scoring system to rank the extent of neurite formation in each condition by assigning each a score from 0 to 8, with 0 indicating a total absence of neurites and 8 indicating long neurites on almost all cells (Table 1).

### Calculation of scores for kinase inhibitor phenotypes

Scores from three blind observers were averaged. The average control score for each individual 96 well plate differed slightly, since some individual plates had slightly longer or shorter neurites in general. For the kinase inhibitor screening experiment, we used eighteen 96 well plates, and there were 8 control wells on each 96 well plate. The mean control phenotype for all 18 of the 96 well plates was 5.04. The average of all controls (5.04) was subtracted from the average score for each plate to determine the amount that the mean scores would be adjusted by in order to normalize the scores. This value was then subtracted from the mean experimental scores for each kinase inhibitor to normalize each score. The following calculation was done to control for the slight differences in neurite length between each individual plate: ((Observer 1 Score + Observer 2 Score + Observer 3 Score)/3) −(Plate Specific Mean Control Score − 5.04) = Normalized Score.

### PBS or valproic acid (VPA) injection

At E9.5 pregnant dams were injected intraperitoneally with 500 mg/kg VPA or equal volume PBS as a control as previously performed^90^. Primary cortical neurons were prepared from the embryos at E14.5, transfected with GFP to visualize morphology, and plated in wells containing coverslips coated with PLL and laminin. After 6 days in vitro, the neurons were fixed using 4% PFA and stained by appropriate antibodies and DAPI. Coverslips were embedded with 90% glycerol made with Tris-buffered saline.

### Aurora and Nuak kinase inhibitors

The inhibitors selected for use in the experiments further investigating the Aurora and Nuak kinases were used in previous publications. These inhibitors are well validated and previous studies have established their ability to block downstream signaling. To validate MK-5108 (VX-689), a panel of seven gene expression pharmacodynamic biomarkers specific for Aurora A inhibition were evaluated in a phase I study^29^. Hesperadin-treated cells were found to have reduced phosphorylation of serine 10 on histone H3 and because H3-Ser10 phosphorylation depends on Aurora B, Hesperadin inhibits Aurora B^28,32^. The ability of TAK-901 to inhibit Aurora A/B has been investigated using Aurora A and B kinase assays to study enzyme activities^24^. GSK1070916 was determined to be a selective inhibitor of Aurora B and Aurora C kinases by using in vitro kinase assays and a reducing in H3-Ser10 phosphorylation was also seen^30^. SNS-314 Mesylate treatment has been shown to correlate with inhibition of H3-Ser10 phosphorylation and inhibition of cell-cycle progression^31^. HTH-01-015 and WZ4003, Nuak1 and Nuak 1/2 inhibitors, respectively, have been shown to inhibit the phosphorylation of MYPT1 (myosin phosphate-targeting subunit 1), which is a well-characterized substrate that is phosphorylated by Nuak Kinase 1^27^. Additionally, we optimized inhibitor dosage to 5 µM for cell health in primary cortical neurons.

### Plasmids

pCAG-mRFP was a gift from Joseph Loturco (Addgene plasmid # 28311; http://n2t.net/addgene:28311; RRID:Addgene_28311). pCAG-YFP was a gift from Connie Cepko (Addgene plasmid # 11180 ; http://n2t.net/addgene:11180; RRID:Addgene_11180). CDNA expression plasmids for Aurora kinases A, B, and C and Nuak kinases 1 and 2 were purchased from OriGene Technologies, Inc., (Rockville, MD). N-terminal Myc/DDK-tagged cDNAs in pCMV6-Entry vector were used. All plasmids were purified using NucleoBond Xtra purification kit (MACHEREY-NAGEL Inc., Bethlehem, PA).

### Antibodies

The primary and secondary antibodies used in this research were as follows: 1:1000 Anti-DYKDDDDK epitope (FLAG) tag (Thermo Scientific Pierce, MA1-91878), 1:5000 HRP-conjugated donkey-anti-mouse IgG, Anti-Aurora Kinase A (abcam, ab13824), Anti-Aurora Kinase B (abcam, ab2254), Anti-Aurora Kinase C (MyBioSource, MBS2032677), Anti-Nuak1 (Proteintech, 22723-1-AP, Anti-Nuak2 (Bioss, bs-5811R), 1:200 Cy5-conjugated donkey-anti-mouse IgG, 1:200 Cy5-conjugated donkey-anti-rabbit IgG.

### Primary mouse cortical neuronal culture and overexpression of Aurora and Nuak kinases

Primary mouse cortical neurons were harvested from embryonic mice at E15.5, prior to gliogenesis at E17.5, to allow for the exclusive culture of neurons^91^. Neurons were plated in wells coated with PLL and laminin. Co-transfection of mRFP + Aurora kinases A, B, and C, and mRFP + Nuak 1 and 2 kinases was performed via chemical transfection using TransIT-X2 (Mirus Bio, Madison, WI) 48 h following plating. Neurons were re-plated on coverslips coated in PLL and laminin and fixed using 4% PFA after an additional 48 h. Re-plating allowed neurite formation to occur after the transfection had taken full effect. Additionally, re-plating of primary neurons is a recognized model for neurite regeneration^35,36^.

### Treatment of primary mouse cortical neurons with Aurora and Nuak kinase inhibitors

Primary mouse cortical neurons were harvested and cultured as described above. Neurons were transfected with YFP via nucleofection. Neurons were treated with Neurobasal media containing 5 µM of kinase inhibitor from the DiscoveryProbe Kinase Inhibitor Library (APExBIO). The following inhibitors were used: MK-5108 (VX-689)—highly selective inhibitor of Aurora Kinase A, Hesperadin—Aurora Kinase B inhibitor, TAK-901—Aurora A/B inhibitor, GSK1070916—Aurora B/C inhibitor, SNS-314 Mesylate—potent and selective inhibitor of Aurora A/B/C kinases, HTH-01-015—highly specific and selective Nuak 1 inhibitor, WZ4003—potent and selective Nuak 1/2 inhibitor. Controls were treated with DMSO, as the inhibitors were diluted in DMSO. Neurons were fixed 48 h after re-plating using 4% PFA.

### Analysis of neuronal morphology

ImageJ software measuring features were used to analyze neuronal morphology of primary cortical neurons. Maximum projection images from z-projection photos produced from z-stack data collected by confocal microscope (Leica SP2 and SP8) were used for analysis. Sholl analysis was performed using the Sholl Analysis Plugin (Gosh Lab, UCSD) for ImageJ following the developer instructions. The longest neurite was considered the neurite most likely to become the axon, while the remaining neurites were considered neurites likely to become dendrites.

### Statistical analysis

Quantitative data were subjected to statistical analysis using SPSS (IBM Analytics). The data were analyzed by one-way ANOVA with Bonferroni when appropriate. Values represented as mean ± SEM. Results were deemed statistically significant if the *p* value was < 0.05.

## Supplementary Information

Supplementary Information 1.

Supplementary Information 2.

Supplementary Information 3.

## Data Availability

See ‘Availability of materials and data’ section for more information.
