# Supplementary material for: High-throughput kinase inhibitor screening reveals roles for Aurora and Nuak kinases in neurite initiation and dendritic branching
Source: Sci Rep. 2021 Apr 14;11:8156. doi: 10.1038/s41598-021-87521-3 (PMC8047044; doi:10.1038/s41598-021-87521-3)

Supplementary Information

High-Throughput Kinase Inhibitor Screening Reveals Roles for Aurora and Nuak Kinases in Neurite Initiation and Dendritic Branching

Sara M. Blazejewski, Sarah A. Bennison, Xiaonan Liu, and Kazuhito Toyo-oka*

Department of Neurobiology and Anatomy, Drexel University College of Medicine, Philadelphia, PA 19129 USA

Corresponding Author*:
Kazuhito Toyo-oka, Ph.D. Email: kt469@drexel.edu

Supplemental Figure 1. **Blazejewski et al.**

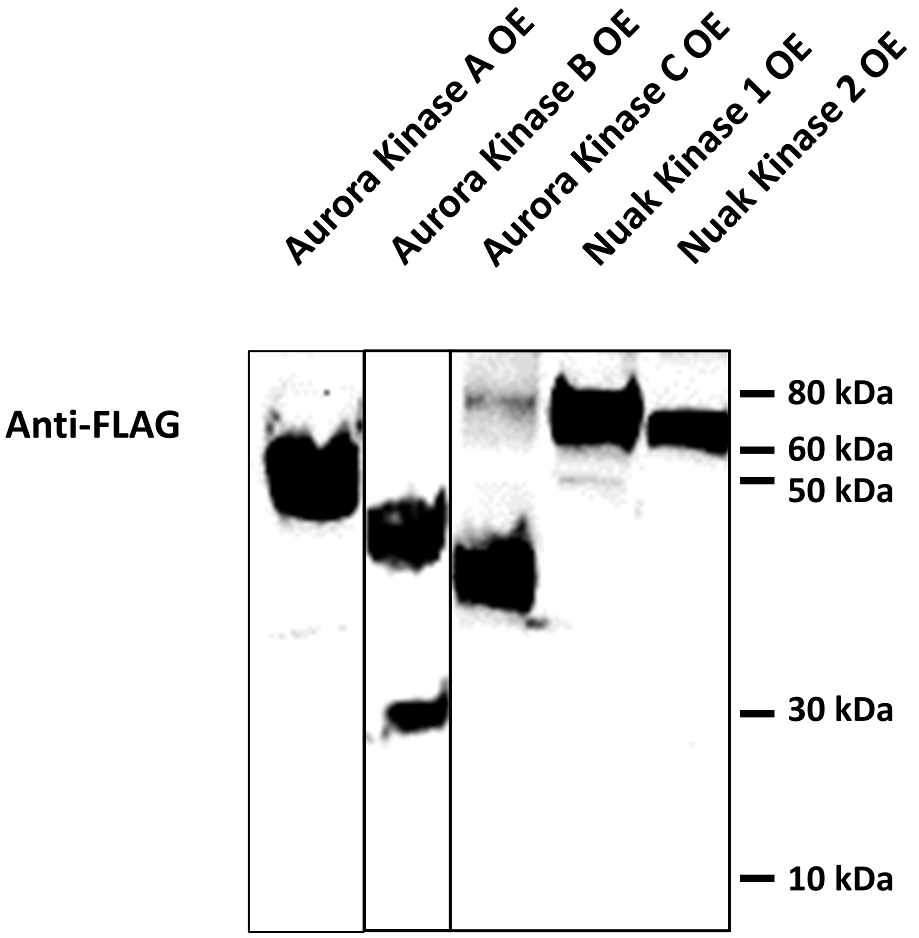

Supplemental Figure 2. **Blazejewski et al.**

Anti-FLAG

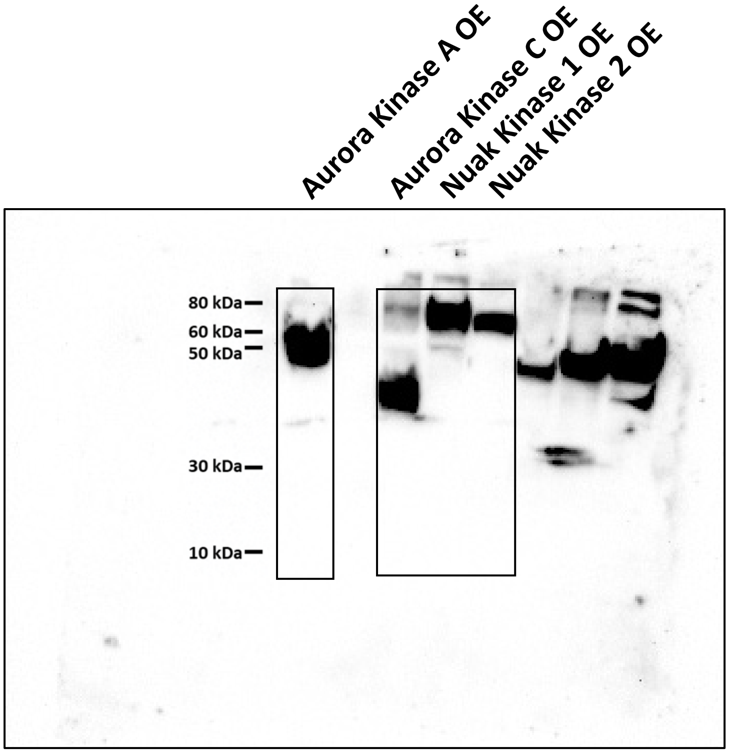

Anti-FLAG

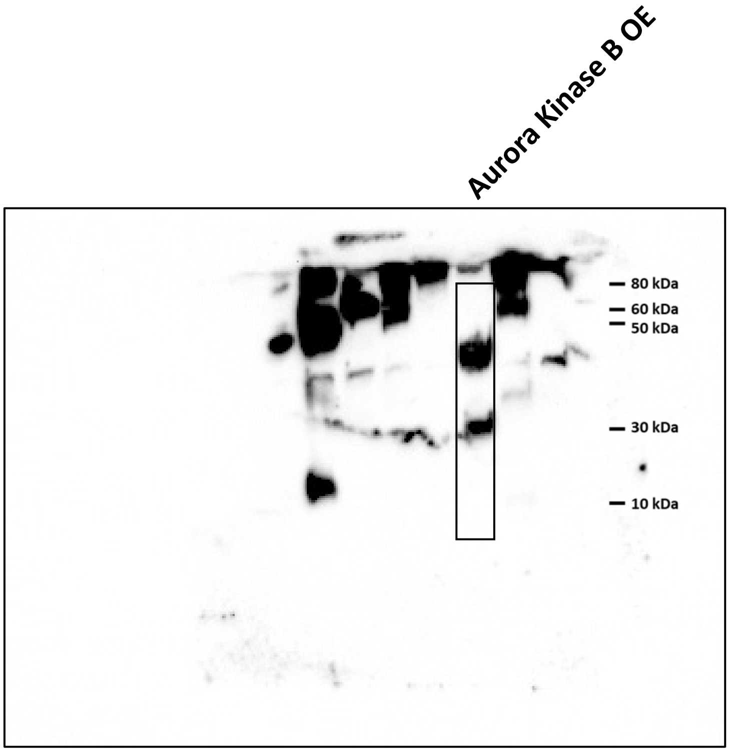

Supplement: Supplementary file 1 — Supplementary Information 1. [file 41598_2021_87521_MOESM1_ESM.pdf]
